# Associations of discrimination and physical activity with social pain sensitivity and a moderating effect of gender in young adults

**DOI:** 10.1371/journal.pone.0333507

**Published:** 2025-10-07

**Authors:** Masataka Umeda, Youngdeok Kim, Se-Woong Park, Eunhee Chung

**Affiliations:** 1 Department of Kinesiology, the University of Texas at San Antonio, San Antonio, Texas, United States of America; 2 Department of Kinesiology and Health Sciences, Virginia Commonwealth University, Richmond, Virginia, United States of America; Erzurum Technical University: Erzurum Teknik Universitesi, TÜRKIYE

## Abstract

**Objective:**

Social pain can be evoked by social rejection. Discrimination is a type of social rejection based on attributes of individuals and linked to pain and hyperalgesia, whereas physical activity (PA) is a healthful behavior that is linked to pain relief and hypoalgesia. However, associations of discrimination and PA with social pain sensitivity is currently unknown. Gender differences in social pain sensitivity exist, but it is unclear if gender moderates the associations of discrimination and PA with social pain sensitivity. The primary aim of this study was to examine the associations of perceived discrimination and PA with social pain sensitivity, and the secondary aim was to explore the moderating effect of gender.

**Methods:**

A total of 172 young adults completed 1) Brief Fear of Negative Evaluation Scale (BFNES) and Social Pain Questionnaire (SPQ) to evaluate social pain sensitivity, 2) Everyday Discrimination Scale (EDS) to quantify perceived discrimination, and 3) PA survey.

**Results:**

The EDS scores were associated with both BFNES and SPQ scores in the total sample (BFNES: *Β *= 0.16, p = 0.028 & SPQ: *Β *= 0.25, p < 0.001). The EDS scores were associated with both BFNES and SPQ scores in men (BFNES: *Β *= 0.28, p = 0.014 & SPQ: *Β *= 0.46, p < 0.001), but not in women (BFNES: *Β *= 0.11, p = 0.221 & SPQ: *Β *= 0.10, p = 0.239). PA levels were associated with the SPQ scores (*Β *= −0.34, p = 0.024) in the total sample, but when stratified by gender, the significant association disappeared for both men and women (men: *Β *= −0.49, p = 0.065 & women: *Β *= −0.33, p = 0.082).

**Conclusions:**

Both perceived discrimination and PA levels were associated with social pain sensitivity. Gender moderated the association between perceived discrimination and social pain sensitivity.

## Introduction

Human pain research has a long history. While majority of the research has examined the sensitivity to various nociceptive physically painful stimuli (e.g., mechanical pressure, etc.) and severity of clinical pain, some research has been conducted to examine social pain, defined as an unpleasant emotional experience evoked by actual or potential damage to one’s sense of social connection and social value [1,2]. The emotional pain, albeit no physical damage to our body, that we may experience when we are rejected from social networks is a common example of how social pain could arise during the daily life. Studies demonstrate a link between physical pain and social pain. For example, those who are more sensitive to acute laboratory physical pain stimuli are more distressed from acute laboratory social rejection experience [3]. Other studies show that social pain sensitivity is associated with physical pain sensitivity [4] and clinical pain intensity [5,6], whereas chronic musculoskeletal pain patients exhibit higher social pain sensitivity compared to healthy controls [7]. These observations collectively suggest that social pain sensitivity could potentially serve as a unique risk factor for nociceptive physical pain and clinical pain.

The most explicit and potentially common social rejection experience that may result in social pain is discrimination. Unfortunately, discrimination happens in many forms in our society based on various attributes of individuals, such as race/ethnicity, sex/gender, nationality, age, sexual orientation, physical/mental disability, etc. Past research shows that perceived discrimination is linked to increased sensitivity to laboratory physical pain stimulus [8], whereas perceived discrimination elevates risks for clinical pain [9–13]. Due to these adverse effects of discrimination, discrimination has been considered as a social determinant of health [14]. Given that discriminated individuals likely experience social pain, it is possible that repeated exposure to discrimination may sensitize the individuals to develop higher sensitivity to social pain. The increased social pain sensitivity may then place the discriminated individuals at elevated risk for physical pain and clinical pain. However, there is currently a paucity of knowledge in the literature regarding the relationship between discrimination and social pain sensitivity.

While discrimination is generally considered as a negative factor for health, physical activity (PA) is known as an established healthful behavior to improve and maintain health [15]. Specifically, research demonstrates that regular PA is associated with decreased sensitivity to laboratory physical pain stimuli [16–18] and contributes to clinical pain relief [19], suggesting a close link of PA to physical pain and clinical pain. In contrast, the relationship between PA and social pain sensitivity is currently unknown. There is some evidence that social pain sensitivity is associated with anxiety and depression [1], and it is well documented that PA produces anxiolytic and antidepressant effects [20,21]. These observations suggest that PA may also be inversely associated with social pain sensitivity, but research is warranted to confirm the association between PA and social pain sensitivity.

Collectively, research suggests that discrimination is positively associated with social pain sensitivity, whereas PA is inversely associated with social pain sensitivity. Given that social pain sensitivity may serve as a unique risk factor for pain, better understanding on the associations of the two factors with social pain sensitivity may contribute to further development of pain prevention and management strategies. On the other hand, research indicates that gender differences exist in pain, such that women are more sensitive to physical pain stimuli and likely to experience clinical pain compared to men [22,23]. In agreement with the research, emerging evidence indicates that gender differences exist in social pain sensitivity as well, with women more sensitive to social pain compared to men [2,24]. If both discrimination and PA are linked to social pain sensitivity and gender differences exist in social pain sensitivity, it would be possible that the strength of the associations of discrimination and PA with social pain sensitivity may differ by gender, such that the positive association of discrimination with social pain sensitivity may be observed more strongly in women compared to men, and/or the inverse association of PA with social pain sensitivity may be observed more strongly in men compared to women. Such gender-moderation of the associations may potentially elevate women’s social pain sensitivity and then underlie the documented sex/gender disparities in pain. It is currently unclear, however, whether gender moderates the associations of discrimination and PA with social pain sensitivity.

Therefore, the primary aim of the present study was to examine the associations of perceived discrimination and PA with social pain sensitivity. We hypothesized that perceived discrimination would be positively associated with social pain sensitivity, whereas PA would be inversely associated with social pain sensitivity. Second, the present study aimed to explore the moderating effect of gender on the associations. We hypothesized that the association of perceived discrimination with social pain sensitivity would be observed more strongly in women compared to men, whereas the association of PA with social pain sensitivity would be observed more strongly in men compared to women.

## Materials and methods

### Participants

Participants in this study were recruited from undergraduate courses in the Department of Kinesiology at a public state university in Texas, US from spring 2023 to spring 2024. Research staff visited several courses to announce the study participation opportunity to the students, and those who were interested in participating in the present study were instructed to visit an online survey platform (Qualtrics.com) to complete several questionnaires independently. The study was fully approved by the Institutional Review Board at the University of Texas at San Antonio (IRB#22-23-32), where the study was conducted, and all participants completed a written consent form online before starting to answer any questions in the survey. A total of 172 students participated in the survey.

### Measures

The present study was a cross-sectional study that aimed to examine the links of discrimination and PA with social pain sensitivity and a moderating effect of gender on the associations. The participants completed the following questionnaires.

#### General information questionnaire.

The general information questionnaire first asked the participants’ demographic and anthropometric information, such as their age, height (cm), weight (kg), sex/gender, and race/ethnicity (American Indian/Alaskan Native, Asian, Black/African American, Hispanic/Latino, Native Hawaiian/Other Pacific Islander, White, and others). Due to the small number of Asian individuals, American Indian/Alaskan Native individuals, Native Hawaiian/Other Pacific Islander individuals, and individuals of other racial/ethnic backgrounds, these individuals were collapsed into one group as others for the analysis. Body mass index (BMI) was calculated using the following formula: BMI = weight (kg)/height (m)^2^ and the participants were categorized into <25 kg/m^2^ and ≥25 kg/m^2^. The questionnaire then asked the participants to indicate how much time they spent for the moderate-to-vigorous intensity aerobic PA (MVPA) and how often they engaged in the muscle-strengthening PA per week as defined by the PA guidelines for adults [15]. The participants chose one response from the six response options that best described how much they normally spent for the MVPA per week (1: not at all, 2: less than 150 minutes, 3: 150 minutes to less than 300 minutes, 4: 300 minutes to less than 450 minutes, 5: 450 minutes to less than 600 minutes, 6: 600 minutes or more), and another one response from the eight response options that best described how often they normally engaged in the muscle-strengthening PA per week (1: not at all, 2: one day, 3: two days, 4: three days, 5: four days, 6: five days, 7: six days, 8: seven days). Those who reported ≥ 150 minutes for MVPA and ≥ two days for muscle-strengthening PA were categorized as sufficiently physically active in each activity category.

#### Brief fear of negative evaluation scale.

The BFNES has been frequently used in past research to evaluate social pain sensitivity [1,2,25] and found to be linked to clinical pain [5–7]. Specifically, the BFNES assesses the degree to which an individual is sensitive to negative evaluation by others, and consists of 12 statements that describe various social interactions with others, including four positively-worded statements and eight negatively-worded statements. For each statement, the participants endorsed one response out of the five response options (1: not at all characteristic of me, 2: slightly characteristic of me, 3: moderately characteristic of me, 4: very characteristic of me, 5: extremely characteristic of me) that best characterized them. After the numeric responses to the negatively-worded statements were reversed, all responses were summed up to represent a total score of the BFNES, with higher scores indicative of being more sensitive to social pain (score range: 12–60). The construct validity of BFNE in the present sample, tested using confirmatory factor analysis (CFA), demonstrated an acceptable model-data fit based on a Root Mean Square Error of Approximation (RMSEA) <0.08, a Comparative Fit Index (CFI) > 0.90, a Tucker-Lewis Index (TLI) > 0.90, and a Standardized Root Mean Squared Residual (SRMR) < 0.08 [26], and internal consistency with a composite reliability score of 0.88 [27] (S1 Table).

#### Social pain questionnaire.

The SPQ is a self-report questionnaire that has been developed more recently to evaluate emotional reaction to social exclusion [28]. The SPQ consists of 10 items that describe various social situations with others that could provoke strong emotional reaction (e.g., I feel very humiliated when I am excluded from a group). For each item, the participants were asked to choose one response out of the five response options that best characterized them (0: applies not at all to me, 1: applies rather not to me, 2: applies in part to me, 3: applies largely to me, 4: applies exactly to me). Responses from the 10 items were then averaged to calculate the SPQ scores, with higher scores indicative of higher social pain sensitivity (score range: 0–4). In the present sample, CFA demonstrated acceptable model-data fit indices (RMSEA = .07; CFI = 0.96; TLI = 0.95; SRMR = 0.05) and composite reliability of.86 (S2 Table).

#### Everyday discrimination scale.

The EDS evaluates how often individuals experience discriminatory treatment during daily life and consists of nine items that describe various interactions with others that could be suggestive of discriminatory treatments (e.g., You are treated with less courtesy than other people are.). For each item, the participants were asked to choose one response out of six response options that best described how often they experienced those treatments during the last 12 months (0: never, 1: less than once a year, 2: a few times a year, 3: a few times a month, 4: at least once a week, 5: almost every day). The numeric responses were summed up to calculate the EDS scores, with the higher EDS scores indicative of more frequent experiences of discriminatory treatments. In the present sample, the model-data fit was acceptable with RMSEA = .08; CFI = 0.96; TLI = 0.94; SRMR = 0.05) and composite reliability of.85 (S3 Table).

After completing the nine items, the participants who chose a 2 (a few times per year) or above to any of the nine items were asked to answer one more question regarding the main reason why they think they were treated so. The participants chose one response out of the ten response options (0: sex/gender, 1: race/ethnicity, 2: age, 3: religion, 4: physical appearance, 5: sexual orientation, 6: education/academic performance, 7: income levels, 8: physical disability, 9: others, including multiple of them) to indicate the most likely reason of the perceived discrimination.

### Data analysis

#### Sample size estimate.

The primary aim of the present study was to examine the associations of perceived discrimination and PA with social pain sensitivity. Due to the lack of pilot data, we reviewed past studies reporting the association of certain psychophysiological variables as independent variables, such as self-construal [24] and blood pressure [1,2], and social pain sensitivity as dependent variable in young adults to estimate an adequate sample size for the present study. The studies were conducted with an average of 128 participants and successfully reported significant associations of the variables with social pain sensitivity, characterized as small-to-medium effect sizes (rs = 0.24–0.58) [1,2,24]. Therefore, a power analysis was performed, with an alpha = 0.05, a power = 0.80, and a small-to-medium effect size assumed, and estimated that approximately 140 participants would be needed for the present study to test the associations of the variables of our primary interest with social pain sensitivity. This sample size would also be adequate to detect gender differences in social pain sensitivity in young adults as the past study showed that approximately 106 healthy adults (53 men and 53 women) would be needed to detect gender differences in social pain sensitivity [24].

#### Analytical approaches.

Descriptive statistics were calculated for the study variables using mean (standard deviation) for continuous variables and frequency (%) for categorical variables. The normality of the variables was assessed using skewness (<± 3.0) and kurtosis (<± 8.0) statistics, along with visual inspection of normal Q-Q plots. Bivariate correlation coefficients were estimated for pairs of study variables. A path model was constructed to examine the associations of EDS and MVPA levels with BFNES and SPQ, while adjusting for study covariates, including age, sex/gender, and race/ethnicity. Follow-up analyses stratified by sex were conducted to estimate sex-specific associations, adjusting for age and race. For all analyses, categorical variables were dummy-coded, where reference groups were selected based on both theoretical and empirical considerations: ‘women’ was used as the reference group for gender based on prior literature indicating greater social and physical pain sensitivity among women [2,22,24]; ‘White’ was selected as the reference for race/ethnicity given large representation in our sample; and ‘insufficient MVPA’ served as the reference for MVPA levels, aligning with current PA guidelines as <150 minutes/week [29]. Results from path analyses are presented as standardized regression coefficients (*β*) with 95% confidence intervals (CIs). Statistical significance was set at *P* < .05 for analysis with total sample, and a Bonferroni-adjusted significance level of <.025 was applied for the gender-stratified path analyses to address the risk of inflated Type I error. Additionally, as a sensitivity analysis, we applied the Benjamini-Hochberg procedure to control the false discovery rate and assess the robustness of the estimates from path analyses. SAS v9.4 was used for data management, and Mplus v7.2 (Muthén & Muthén, Los Angeles, CA) was used for path analyses.

## Results

Descriptive statistics of the study sample are presented in Table 1. Of the 172 participants, 65.70% (*N* = 113) were women. Men showed a greater tendency to report sufficient MVPA levels (79.66%) compared to women (65.49%; *P* = .053). Women reported significantly higher social pain sensitivity scores (BFNES: 35.90 ± 9.57; SPQ: 2.04 ± 0.70) compared to men (BFNES: 31.39 ± 6.90; SPQ: 1.71 ± 0.74); however, no significant gender differences were observed for the EDS scores (*P* = .812). A descriptive analysis on potential reasons for the perceived discrimination revealed that among those who reported being discriminated during the last 12 months (*N* = 128), physical appearance, sex/gender, race/ethnicity, age, education/academic performance, and religion were single reasons for the discrimination. However, 27 participants indicated that other reasons, including more than one of those reasons, were why they experienced the discrimination. Another single reason for the discrimination included “being an athlete”, “personality”, “attitude”, and “job”. In contrast, several participants attributed those who discriminated them (e.g., their mindset, etc.) to their discriminatory experiences. These data are summarized in Table 2.

**Table 1:** Descriptive characteristics of the study sample by gender.

	Total	Men	Women	*P*-value^a^
n	172 (100%)	59 (34.30%)	113 (65.70%)	
Age (years)	22.08 (2.83)	22.58 (3.05)	21.82 (2.68)	.098
Race (n, %)				.344
White	36 (20.93%)	10 (16.95%)	26 (23.01%)	
Black	20 (11.63%)	10 (16.95%)	10 (8.85%)	
Hispanic & Latino	96 (55.81%)	31 (52.54%)	65 (57.52%)	
Others	20 (11.63%)	8 (13.56%)	12 (10.62%)	
MVPA levels^b^				.053
Sufficient	121 (70.35%)	47 (79.66%)	74 (65.49%)	
Insufficient	51 (29.65%)	12 (20.34%)	39 (34.51%)	
Social pain				
BFNES scores	34.35 (8.99)	31.39 (6.90)	35.90 (9.57)	<.001
SPQ scores	1.93 (0.73)	1.71 (0.74)	2.04 (0.70)	.005
Discrimination history				
EDS scores	12.95 (7.91)	13.15 (9.00)	12.85 (7.32)	.812

BFNES = Brief Fear of Negative Evaluation Scale; EDS = Everyday Discrimination Scale; MVPA = moderate and vigorous intensity physical activity; SPQ = Social Pain Questionnaire.

the estimates are frequency (%) for categorical variables and mean (standard deviation) for continuous variables.

^a^*P*-value is obtained from independent sample t-test and chi-square test of independence for continuous and categorical variables, respectively.

^b^MVPA level was categorized as sufficient based on the self-reported MVPA minutes per week (Sufficient: ≥ 150 minutes/week & Insufficient: < 150 minutes/week).

**Table 2:** Potential reasons for discrimination.

Reasons for discrimination	Frequency (%)
Physical appearance (e.g., height, weight, etc.)	31 (18.02)
Sex/gender	24 (13.95)
Race/ethnicity	20 (11.63)
Age	13 (7.56)
Education/academic performance	7 (4.07)
Religion	6 (3.49)
Sexual orientation	0
Income levels	0
Physical disability	0
Others (including more than one reason above)	27 (15.70)
Not applicable	44 (25.58)
	Total 172 (100)

Table 3 presents the bivariate correlation coefficients between study variables. The EDS scores were positively correlated with the BFNES (*r* = 0.149, *P* = .052) and SPQ scores (*r* = 0.252, *P* < .001), whereas MVPA was negatively correlated with the BFNES (*r* = −0.152, *P* = .047) and SPQ scores (*r* = −0.168, *P* = .028).

**Table 3:** Correlation matrix between study variables (*N* = 172).

	BFNES	SPQ	EDS	Age	Sex	Race (Black)	Race (H&L)	Race (Others)	MVPA (Sufficient)
BFNES	–	–	–	–	–	–	–	–	–
SPQ	0.600^*^	–	–	–	–	–	–	–	–
EDS	0.149	0.252^*^	–	–	–	–	–	–	–
Age	−0.249^*^	−0.260^*^	−0.083	–	–	–	–	–	–
Sex (Men)	−0.239^*^	−0.219^*^	0.018	0.127	–	–	–	–	–
Race (Black)	−0.099	−0.100	0.0001	0.067	0.120	–	–	–	–
Race (Hispanic & Latino)	0.064	0.010	−0.171^*^	−0.107	−0.048	−0.408^*^	–	–	–
Race (Others)	0.020	0.005	−0.014	0.028	0.044	−0.132	−0.408^*^	–	–
MVPA (Sufficient)	−0.152^*^	−0.168^*^	0.077	0.001	0.147	0.116	−0.039	−0.082	–

BFNES = Brief Fear of Negative Evaluation Scale; EDS = Everyday Discrimination Scale; MVPA = moderate and vigorous intensity physical activity; SPQ = Social Pain Questionnaire.

*P < .05.

**Fig 1:**
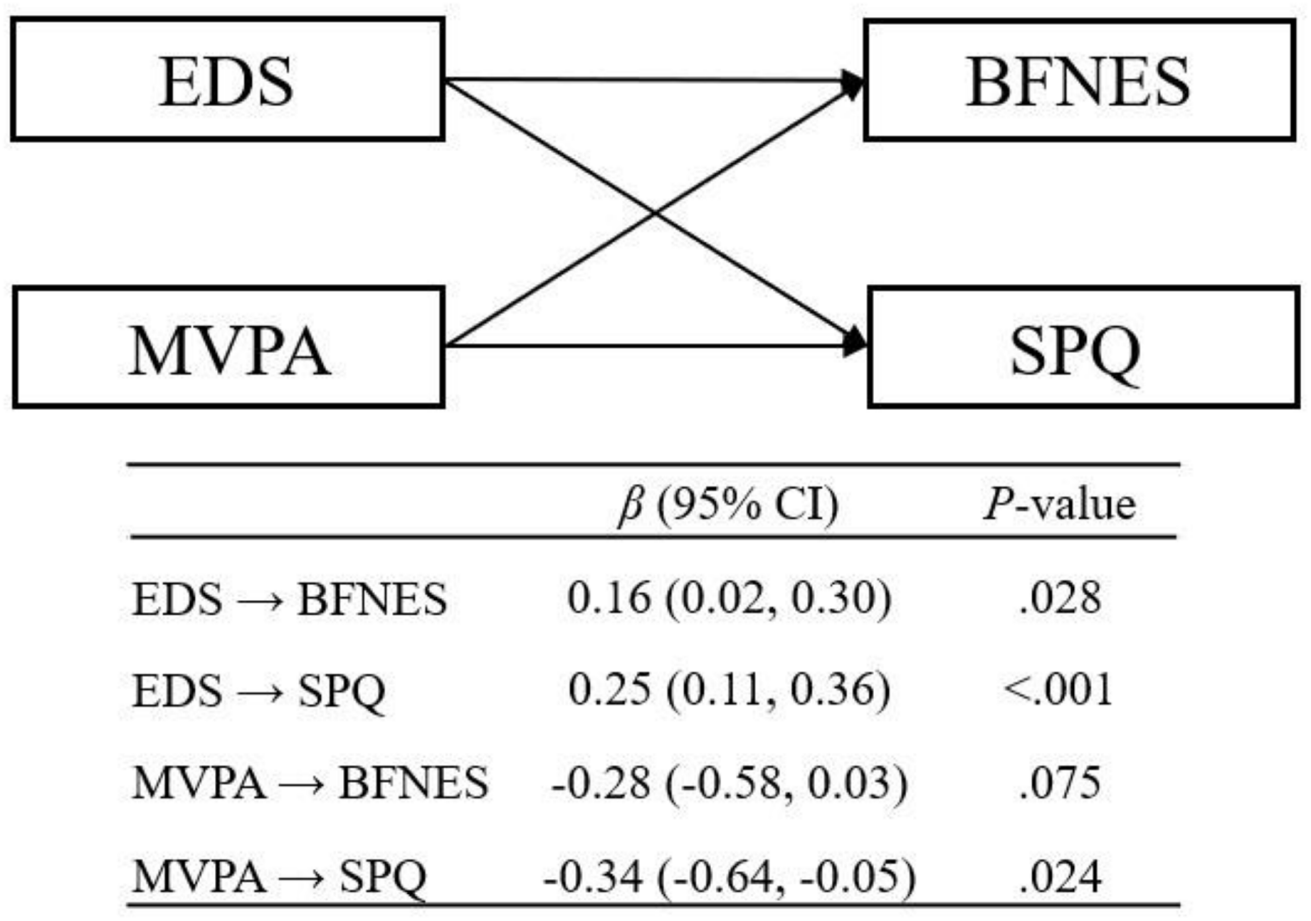
Path diagram examining the relationship of Everyday Discrimination Scale (EDS) and MVPA levels (insufficient vs. sufficient) with the Brief Fear of Negative Evaluation Scale (BFNES) and the Social Pain Questionnaire (SPQ). The paths from study covariates including age (years), gender (men vs. women), and race (White vs. Black; White vs. Hispanic/Latino; White vs. Others) are omitted for simplicity. The main results are presented in the table below as standardized regression coefficients with 95% confidence intervals.

Results from the path analysis based on the total sample are presented in Fig 1. After adjusting for study covariates, including age, sex, and race, the EDS scores were significantly associated with the BFNES (*β* = 0.16, *P* = .028) and SPQ scores (*β* = 0.25, *P* < .001). A significant association between MVPA levels and SPQ scores was observed (*β* = −0.28, *P* = .024), but not with the BFNES scores (*β* = −0.28, *P* = .075). In the follow-up gender-stratified analyses (Table 4), significant associations of the EDS scores with the BFNES and SPQ scores were retained only in men (BFNES: *β* = 0.28, *P* = .014; SPQ: *β* = 0.46, *P* < .001) but not in women (BFNES: *β* = 0.11, *P* = .221; SPQ: *β* = 0.10, *P* = .239). The MVPA levels were no longer significant predictors of the SPQ scores in either men (*β* = −0.49, *P* = .065) or women (*β* = −0.33, *P* = .082). All significant associations identified in the path analyses remained significant after applying the Benjamini-Hochberg procedure to control the false discovery rate, supporting the robustness of the estimates.

**Table 4:** Results of path analysis stratified by gender.

	Men		Women	
	*β* (95% CI)	*P*-value	*β* (95% CI)	*P*-value
EDS → BFNES	0.28 (0.05, 0.51)	.014	0.11 (−0.07, 0.30)	.221
EDS → SPQ	0.46 (0.26, 0.66)	<.001	0.10 (−0.07, 0.29)	.239
MVPA → BFNES	−0.54 (−1.10, 0.02)	.059	−0.24 (−0.62, 0.14)	.222
MVPA → SPQ	−0.48 (−0.99, 0.03)	.065	−0.33 (−0.70, 0.04)	.082

BFNES = Brief Fear of Negative Evaluation Scale; EDS = Everyday Discrimination Scale; MVPA = moderate and vigorous intensity physical activity; SPQ = Social Pain Questionnaire

The estimated are presented as standardized regression coefficients with 95% confidence intervals, adjusted for study covariates presented in Table 1. A Bonferroni-adjusted alpha level of 0.025 was applied to determine statistical significance.

## Discussion

The primary aim of the present study was to examine the associations of perceived discrimination and PA with social pain sensitivity, a unique risk factor for pain, in young adults who were recruited from college courses. Second, the present study aimed to explore a moderating effect of gender on the associations. The results indicated that many participants reported being discriminated due to various reasons, such as physical appearance, sex/gender, race/ethnicity, age, education/academic performance, and religion. Their perceived discrimination was then positively associated with social pain sensitivity, whereas PA was inversely associated with social pain sensitivity. Next, the gender-stratified analysis indicated that gender moderated the association between perceived discrimination and social pain sensitivity, such that the association was observed only in men, but not in women, whereas gender did not moderate the association between PA and social pain sensitivity. Together, the present study added to the literature by showing that perceived discrimination and lack of PA contribute to the development of higher social pain sensitivity, and gender plays a moderating role in the association between perceived discrimination and social pain sensitivity.

The present study indicated that perceived discrimination was positively associated with social pain sensitivity in a total sample of young adults, whereas PA was inversely associated with social pain sensitivity. The findings supported our hypothesis and were conceptually in agreement with past research that perceived discrimination is positively associated with laboratory physical pain sensitivity [8] and serves as a risk factor for clinical pain [9–13]. On the other hand, PA is inversely associated with laboratory pain sensitivity [16–18], whereas PA plays a role in management of clinical pain [19]. Collectively, the findings revealed that perceived discrimination and PA influence social pain sensitivity in the similar manners to how perceived discrimination and PA influence physical pain and clinical pain.

The findings that discrimination and PA were associated with social pain sensitivity in the similar manners to physical pain sensitivity further support the previous research that demonstrates the connection between physical pain and social pain. There is evidence that those who are more sensitive to acute laboratory physical pain stimuli are more distressed from acute laboratory social rejection experience [3]. Additionally, past research indicates that social pain sensitivity is associated with physical pain sensitivity [4] and clinical pain severity [5,6], whereas chronic musculoskeletal pain patients show higher social pain sensitivity compared to healthy controls [7]. The connection between physical pain and social pain could be explained by neurophysiological evidence that both physical pain and social pain activate the dorsal anterior cingulate cortex [30,31], whereas resting blood pressure is inversely associated with physical pain [32,33] and social pain sensitivity [1,2]. Furthermore, pharmacological studies show that several pain medications, such as acetaminophen [34,35], oxytocin [36], and opioid [37], could ease social pain as well. Together, results from this study show that the two types of pain may share various correlates and add to the literature regarding the connection between physical pain and social pain.

Furthermore, the present study showed that gender differences were observed in social pain sensitivity, with women more sensitive to social pain compared to men, consistent with past research [2,24]. Additionally, the results showed that gender moderated the association between perceived discrimination and social pain sensitivity, with the association observed only in men, but gender did not moderate the association between PA and social pain sensitivity. The findings did not support our hypothesis as we hypothesized that gender would play a moderating role in the associations, such that the association between perceived discrimination and social pain sensitivity would be observed more strongly in women, whereas the association between PA and social pain sensitivity would be observed more strongly in men. The hypothesis was based upon the theoretical assumption that both perceived discrimination and PA were the factors that would contribute to gender differences in social pain sensitivity; however, the fact that the present study also demonstrated gender differences in social pain sensitivity potentially suggests that perceived discrimination and PA contribute to the development of social pain sensitivity, but do not underlie gender differences in social pain sensitivity. More research is needed to understand what underlies gender differences in social pain sensitivity.

The present study has several limitations. First, the study sample consisted of young adults who were recruited from college courses, which limits generalizability of the findings to other samples (e.g., older adults, individuals with chronic pain, individuals of various socioeconomic status, etc.). Second, the sample size was estimated to test the associations of discrimination and PA with social pain sensitivity based on the past studies with young adults that examined qualitatively different variables of interest (e.g., self-construal [24] and blood pressure [1,2]) due to the lack of pilot data. This approach was taken as an alternative method but may not have been the ideal approach for the sample size estimate. Furthermore, testing the secondary aim (gender moderation of the associations) was likely limited by insufficient statistical power to test the aim. Third, the primary variables of this study, such as discrimination, PA, and social pain sensitivity, were evaluated using the self-report instruments, which are known to be impacted by recall bias and social desirability. Future studies can be strengthened by incorporating an objective assessment of PA and/or laboratory induction of social pain into the research design. Fourth, the present study was conducted in a cross-sectional design, making the temporal sequence and causality of the associations unclear. Lastly, due to relatively small sample size for each reason for perceived discrimination, we did not analyze the potential impact of various reasons for the association with social pain sensitivity. Future research should examine whether strength of the association may vary by the reasons for discrimination. Together, results from this study should be interpreted with caution due to these methodological limitations.

In conclusion, the present study examined the links of perceived discrimination and PA with social pain sensitivity in young adults and found that perceived discrimination was positively associated with social pain sensitivity, whereas PA was inversely associated with social pain sensitivity. Furthermore, the association between perceived discrimination and social pain sensitivity was moderated by gender, such that the association was observed only in men, but not in women. The association between PA and social pain sensitivity, on the other hand, was not moderated by gender. More research is warranted to further examine what variables influences social pain sensitivity and moderates the associations.

## Supporting information

S1 Table The Results of Confirmatory Factor Analysis for Brief Fear of Negative Evaluation Scale (BFNES). (DOCX)

S2 Table The Results of Confirmatory Factor Analysis for Social Pain Questionnaire (SPQ). (DOCX)

S3 Table The Results of Confirmatory Factor Analysis for Everyday Discrimination Scale (EDS). (DOCX)

S1 File Minimal Dataset. (XLSX)

## References

[1] Umeda M, Leutze TM, Inagaki TK. Replication and extension of the link between the cardiovascular system and sensitivity to social pain in healthy adults. Soc Neurosci. 2021;16(3):265–76. doi: 10.1080/17470919.2021.1897672 33648414

[2] Inagaki TK, Jennings JR, Eisenberger NI, Gianaros PJ. Taking rejection to heart: associations between blood pressure and sensitivity to social pain. Biol Psychol. 2018;139:87–95. doi: 10.1016/j.biopsycho.2018.10.007 30352273 PMC6295662

[3] Canaipa R, Castro-Caldas A, Moreira JM, Pimentel-Santos F, Branco JC, Treister R. Impaired pain modulation in fibromyalgia patients in response to social distress manipulation. Clin J Pain. 2017;33(7):611–9. doi: 10.1097/AJP.0000000000000447 27841833

[4] Umeda M, Kim Y. The role of anxiety in the association between physical pain and social pain sensitivity in young adults. Pain Manag. 2025;15(5):259–64. doi: 10.1080/17581869.2025.2508680 40392048 PMC12118422

[5] Geelen CC, Smeets RJEM, Schmitz S, van den Bergh JP, Goossens MEJB, Verbunt JA. Anxiety affects disability and quality of life in patients with painful diabetic neuropathy. Eur J Pain. 2017;21(10):1632–41. doi: 10.1002/ejp.1067 28656745

[6] Ecija C, Catala P, Gutierrez L, Javier Arrayás-Grajera M, Peñacoba C. The influence of the fear of negative evaluation on activity avoidance in fibromyalgia: exploring pain acceptance and positive affect as resilience variables. Clin Nurs Res. 2023;32(5):902–13. doi: 10.1177/10547738221122670 36217962

[7] Turner-Cobb JM, Michalaki M, Osborn M. Self-conscious emotions in patients suffering from chronic musculoskeletal pain: a brief report. Psychol Health. 2015;30(4):495–501. doi: 10.1080/08870446.2014.991735 25420522

[8] Richardson EJ, Trost Z, Payne M, Wiggins A. The negative effect of social discrimination on pain tolerance and the moderating role of pain catastrophizing. J Clin Psychol Med Settings. 2023;30(1):169–81. doi: 10.1007/s10880-022-09860-1 35244822

[9] Vang ZM, Chau S, Kobayashi K, Owen MJ, McKenzie-Sampson S, Mayrand-Thibert J, et al. Pain and functional limitations among midlife and older canadians: the role of discrimination, race and sense of belonging. J Gerontol B Psychol Sci Soc Sci. 2021;:gbab137. doi: 10.1093/geronb/gbab137 34282848

[10] Dugan SA, Lewis TT, Everson-Rose SA, Jacobs EA, Harlow SD, Janssen I. Chronic discrimination and bodily pain in a multiethnic cohort of midlife women in the Study of Women’s Health Across the Nation. Pain. 2017;158(9):1656–65. doi: 10.1097/j.pain.0000000000000957 28753588 PMC5561511

[11] Edwards RR. The association of perceived discrimination with low back pain. J Behav Med. 2008;31(5):379–89. doi: 10.1007/s10865-008-9160-9 18581224

[12] Brown TT, Partanen J, Chuong L, Villaverde V, Chantal Griffin A, Mendelson A. Discrimination hurts: the effect of discrimination on the development of chronic pain. Soc Sci Med. 2018;204:1–8. doi: 10.1016/j.socscimed.2018.03.015 29549869

[13] Gee GC, Spencer MS, Chen J, Takeuchi D. A nationwide study of discrimination and chronic health conditions among Asian Americans. Am J Public Health. 2007;97(7):1275–82. doi: 10.2105/AJPH.2006.091827 17538055 PMC1913081

[14] US Department of Health and Human Services OP a HP. Healthy People 2030. [Accessed 2024 January 9]. https://health.gov/healthypeople/objectives-and-data/social-determinants-health

[15] US Department of Health and Human Services. The 2018 Physical Activity Guidelines Advisory Committee Scientific Report. US Department of Health and Human Services. [Accessed 2018 June 13]. https://health.gov/paguidelines/second-edition/report.aspx

[16] Ellingson LD, Colbert LH, Cook DB. Physical activity is related to pain sensitivity in healthy women. Med Sci Sports Exerc. 2012;44(7):1401–6. doi: 10.1249/MSS.0b013e318248f648 22217571

[17] Johnson MH, Stewart J, Humphries SA, Chamove AS. Marathon runners’ reaction to potassium iontophoretic experimental pain: pain tolerance, pain threshold, coping and self-efficacy. Eur J Pain. 2012;16(5):767–74. doi: 10.1002/j.1532-2149.2011.00059.x 22337477

[18] Leźnicka K, Starkowska A, Tomczak M, Cięszczyk P, Białecka M, Ligocka M, et al. Temperament as a modulating factor of pain sensitivity in combat sport athletes. Physiol Behav. 2017;180:131–6. doi: 10.1016/j.physbeh.2017.08.018 28844852

[19] Geneen LJ, Moore RA, Clarke C, Martin D, Colvin LA, Smith BH. Physical activity and exercise for chronic pain in adults: an overview of Cochrane Reviews. Cochrane Database Syst Rev. 2017;1(1):CD011279. doi: 10.1002/14651858.CD011279.pub2 28087891 PMC6469540

[20] Jayakody K, Gunadasa S, Hosker C. Exercise for anxiety disorders: systematic review. Br J Sports Med. 2014;48(3):187–96. doi: 10.1136/bjsports-2012-091287 23299048

[21] Schuch FB, Vancampfort D, Richards J, Rosenbaum S, Ward PB, Stubbs B. Exercise as a treatment for depression: a meta-analysis adjusting for publication bias. J Psychiatr Res. 2016;77:42–51. doi: 10.1016/j.jpsychires.2016.02.023 26978184

[22] Fillingim RB, King CD, Ribeiro-Dasilva MC, Rahim-Williams B, Riley JL 3rd. Sex, gender, and pain: a review of recent clinical and experimental findings. J Pain. 2009;10(5):447–85. doi: 10.1016/j.jpain.2008.12.001 19411059 PMC2677686

[23] Osborne NR, Davis KD. Sex and gender differences in pain. Int Rev Neurobiol. 2022;164:277–307. doi: 10.1016/bs.irn.2022.06.013 36038207

[24] Umeda M, Park S-W. Association between self-construals, social pain sensitivity, and gender in young adults. J Psychol. 2024;158(8):650–65. doi: 10.1080/00223980.2024.2340633 38652651

[25] Inagaki TK, Gianaros PJ. Resting (Tonic) blood pressure is associated with sensitivity to imagined and acute experiences of social pain: evidence from three studies. Psychol Sci. 2022;33(6):984–98. doi: 10.1177/09567976211061107 35613456 PMC9343892

[26] Hu L, Bentler PM. Cutoff criteria for fit indexes in covariance structure analysis: conventional criteria versus new alternatives. Struct Equation Model. 1999;6(1):1–55. doi: 10.1080/10705519909540118

[27] Dash G, Paul J. CB-SEM vs PLS-SEM methods for research in social sciences and technology forecasting. Technol Forecast Soc Change. 2021;173:121092. doi: 10.1016/j.techfore.2021.121092

[28] Stangier U, Schüller J, Brähler E. Development and validation of a new instrument to measure social pain. Sci Rep. 2021;11(1):8283. doi: 10.1038/s41598-021-87351-3 33859226 PMC8050222

[29] Piercy KL, Troiano RP, Ballard RM, Carlson SA, Fulton JE, Galuska DA, et al. The physical activity guidelines for Americans. JAMA. 2018;320(19):2020–8. doi: 10.1001/jama.2018.14854 30418471 PMC9582631

[30] Eisenberger NI. Social pain and the brain: controversies, questions, and where to go from here. Annu Rev Psychol. 2015;66:601–29. doi: 10.1146/annurev-psych-010213-115146 25251482

[31] Eisenberger NI. The pain of social disconnection: examining the shared neural underpinnings of physical and social pain. Nat Rev Neurosci. 2012;13(6):421–34. doi: 10.1038/nrn3231 22551663

[32] Ghione S. Hypertension-associated hypalgesia. Evidence in experimental animals and humans, pathophysiological mechanisms, and potential clinical consequences. Hypertension. 1996;28(3):494–504. doi: 10.1161/01.hyp.28.3.494 8794839

[33] Bruehl S, Chung OY. Interactions between the cardiovascular and pain regulatory systems: an updated review of mechanisms and possible alterations in chronic pain. Neurosci Biobehav Rev. 2004;28(4):395–414. doi: 10.1016/j.neubiorev.2004.06.004 15341037

[34] Dewall CN, Macdonald G, Webster GD, Masten CL, Baumeister RF, Powell C, et al. Acetaminophen reduces social pain: behavioral and neural evidence. Psychol Sci. 2010;21(7):931–7. doi: 10.1177/0956797610374741 20548058

[35] Slavich GM, Shields GS, Deal BD, Gregory A, Toussaint LL. Alleviating social pain: a double-blind, randomized, placebo-controlled trial of forgiveness and acetaminophen. Ann Behav Med. 2019;53(12):1045–54. doi: 10.1093/abm/kaz015 31050715 PMC6845385

[36] Zhang X, Li P, Otieno SCSA, Li H, Leppänen PHT. Oxytocin reduces romantic rejection-induced pain in online speed-dating as revealed by decreased frontal-midline theta oscillations. Psychoneuroendocrinology. 2021;133:105411. doi: 10.1016/j.psyneuen.2021.105411 34537623

[37] Hsu DT, Sanford BJ, Meyers KK, Love TM, Hazlett KE, Wang H, et al. Response of the μ-opioid system to social rejection and acceptance. Mol Psychiatry. 2013;18(11):1211–7. doi: 10.1038/mp.2013.96 23958960 PMC3814222

