# Supplementary material for: Associations of discrimination and physical activity with social pain sensitivity and a moderating effect of gender in young adults
Source: PLoS One. 2025 Oct 7;20(10):e0333507. doi: 10.1371/journal.pone.0333507 (PMC12503236; doi:10.1371/journal.pone.0333507)
Supplement: S1 Table — (DOCX) [file pone.0333507.s001.docx]

Supporting Information Table 1.

The Results of Confirmatory Factor Analysis for Brief Fear of Negative Evaluation Scale (BFNES)

|  | Factor loading | SE | *P*-value | Composite Reliability |
| --- | --- | --- | --- | --- |
| *Survey Items* |  |  |  | 0.879 |
| BFNES#1 | 0.789 | .034 | <.001 |  |
| BFNES#3 | 0.718 | .041 | <.001 |  |
| BFNES#5 | 0.804 | .032 | <.001 |  |
| BFNES#6 | 0.736 | .040 | <.001 |  |
| BFNES#8 | 0.764 | .036 | <.001 |  |
| BFNES#9 | 0.682 | .045 | <.001 |  |
| BFNES#11 | 0.796 | .033 | <.001 |  |
| BFNES#12 | 0.684 | .045 | <.001 |  |
| BFNES#2 | 0.185 | .078 | <.001 |  |
| BFNES#4 | 0.269 | .075 | <.001 |  |
| BFNES#7 | 0.479 | .063 | <.001 |  |
| BFNES#10 | 0.285 | .074 | <.001 |  |
| *Covariance allowed* |  |  |  |  |
| BFNES#6 with BFNES#5 | 0.420 | .073 | <.001 |  |
| BFNES#4 with BFNES#2 | 0.331 | .068 | <.001 |  |
| *Goodness of fit indices* |  |  |  |  |
| *x2 (df)* = 112.28 (52)^*^; RMSEA = 0.082; CFI = 0.934; TLI = 0.916; SRMR = 0.057 | | | | |

SE = standard error; RMSEA = Root Mean Square Error of Approximation; CFI = comparative fit index; TLI = Tucker-Lewis Index; SRMR = standardized root mean squared residual.

^*^ *P*<.05
