# Supplementary material for: Associations of discrimination and physical activity with social pain sensitivity and a moderating effect of gender in young adults
Source: PLoS One. 2025 Oct 7;20(10):e0333507. doi: 10.1371/journal.pone.0333507 (PMC12503236; doi:10.1371/journal.pone.0333507)
Supplement: S2 Table — (DOCX) [file pone.0333507.s002.docx]

Supporting Information Table 2.

The Results of Confirmatory Factor Analysis for Social Pain Questionnaire (SPQ)

|  | Factor loading | SE | *P*-value | Composite Reliability |
| --- | --- | --- | --- | --- |
| *Survey Items* |  |  |  | 0.863 |
| SPQ#1 | 0.610 | .054 | <.001 |  |
| SPQ#2 | 0.747 | .041 | <.001 |  |
| SPQ#3 | 0.791 | .037 | <.001 |  |
| SPQ#4 | 0.643 | .051 | <.001 |  |
| SPQ#5 | 0.649 | .051 | <.001 |  |
| SPQ#6 | 0.618 | .054 | <.001 |  |
| SPQ#7 | 0.543 | .060 | <.001 |  |
| SPQ#8 | 0.646 | .052 | <.001 |  |
| SPQ#9 | 0.563 | .059 | <.001 |  |
| SPQ#10 | 0.376 | .072 | <.001 |  |
| *Covariance allowed* |  |  |  |  |
| SPQ#10 with SPQ#9 | 0.410 | .063 | <.001 |  |
| SPQ#7 with SPQ#8 | 0.375 | .071 | <.001 |  |
| SPQ#9 with SPQ#1 | 0.261 | .069 | <.001 |  |
| *Goodness of fit indices* |  |  |  |  |
| *x*^2^ (^df^) = 56.61 (32)^*;^ RMSEA = 0.067; CFI = 0.961; TLI = 0.945; SRMR = 0.047 | | | | |

SE = standard error; RMSEA = Root Mean Square Error of Approximation; CFI = comparative fit index; TLI = Tucker-Lewis Index; SRMR = standardized root mean squared residual.

^*^ *P*<.05
