# Supplementary material for: Associations of discrimination and physical activity with social pain sensitivity and a moderating effect of gender in young adults
Source: PLoS One. 2025 Oct 7;20(10):e0333507. doi: 10.1371/journal.pone.0333507 (PMC12503236; doi:10.1371/journal.pone.0333507)
Supplement: S3 Table — (DOCX) [file pone.0333507.s003.docx]

Supporting Information Table 3.

The Results of Confirmatory Factor Analysis for Everyday Discrimination Scale (EDS)

|  | Factor loading | SE | *P*-value | Composite Reliability | |
| --- | --- | --- | --- | --- | --- |
| *Survey Items* |  |  |  | 0.852 | |
| EDS#1 | 0.674 | .050 | <.001 |  | |
| EDS#2 | 0.690 | .047 | <.001 |  | |
| EDS#3 | 0.631 | .054 | <.001 |  | |
| EDS#4 | 0.816 | .038 | <.001 |  | |
| EDS#5 | 0.572 | .058 | <.001 |  | |
| EDS#6 | 0.585 | .057 | <.001 |  | |
| EDS#7 | 0.695 | .047 | <.001 |  | |
| EDS#8 | 0.494 | .069 | <.001 |  | |
| EDS#9 | 0.429 | .069 | <.001 |  | |
| *Covariance allowed* |  |  |  |  | |
| EDS#1 with EDS#2 | 0.665 | .047 | <.001 |  | |
| EDS#9 with EDS#5 | 0.327 | .069 | <.001 |  | |
| EDS#9 with EDS#8 | 0.242 | 0.073 | <.001 |  | |
| *Goodness of fit indices* |  |  |  |  | |
| *x2 (df)* = 48.43 (23)^*^; RMSEA = 0.080; CFI = 0.960; TLI = 0.938; SRMR = 0.049 | | | | |  |

SE = standard error; RMSEA = Root Mean Square Error of Approximation; CFI = comparative fit index; TLI = Tucker-Lewis Index; SRMR = standardized root mean squared residual.

^*^ *P*<.05
